# High-Hole-Mobility Metal–Organic Framework as Dopant-Free Hole Transport Layer for Perovskite Solar Cells

**DOI:** 10.1186/s11671-021-03643-7

**Published:** 2022-01-04

**Authors:** Ruonan Wang, Weikang Yu, Cheng Sun, Kashi Chiranjeevulu, Shuguang Deng, Jiang Wu, Feng Yan, Changsi Peng, Yanhui Lou, Gang Xu, Guifu Zou

**Affiliations:** 1 College of Energy, Soochow Institute for Energy and Materials Innovations, and Key Laboratory of Advanced Carbon Materials and Wearable Energy Technologies of Jiangsu Province, Soochow University, Suzhou, 215123 People’s Republic of China; 2 School of Resources Environmental and Chemical Engineering, Nanchang University, 999 Xuefu Avenue, Nanchang, 330031 China; 3 State Key Laboratory of Structural Chemistry, Fujian Institute of Research on the Structure of Matter, Chinese Academy of Sciences, Fuzhou, 350002 Fujian China; 4 School for Engineering of Matter, Transport and Energy, Arizona State University, 551 E. Tyler Mall, Tempe, AZ 85287 USA; 5 Institute of Fundamental and Frontier Sciences, University of Electronic Science and Technology of China, Chengdu, 610054 People’s Republic of China; 6 College of Chemistry, Chemical Engineering and Materials Science, Soochow Universit, Suzhou, 215123 People’s Republic of China; 7 School of Optoelectronic Science and Engineering and Collaborative Innovation Center of Suzhou Nano Science and Technology, Soochow University, Suzhou, 215006 People’s Republic of China

**Keywords:** Dopant-free hole transport materials, Metal–organic frameworks, Perovskite solar cells, High hole mobility, Ni_3_(2,3,6,7,10,11-hexaiminotriphenylene)_2_

## Abstract

A dopant-free hole transport layer with high mobility and a low-temperature process is desired for optoelectronic devices. Here, we study a metal–organic framework material with high hole mobility and strong hole extraction capability as an ideal hole transport layer for perovskite solar cells. By utilizing lifting-up method, the thickness controllable floating film of Ni_3_(2,3,6,7,10,11-hexaiminotriphenylene)_2_ at the gas–liquid interface is transferred onto ITO-coated glass substrate. The Ni_3_(2,3,6,7,10,11-hexaiminotriphenylene)_2_ film demonstrates high compactness and uniformity. The root-mean-square roughness of the film is 5.5 nm. The ultraviolet photoelectron spectroscopy and the steady-state photoluminescence spectra exhibit the Ni_3_(HITP)_2_ film can effectively transfer holes from perovskite film to anode. The perovskite solar cells based on Ni_3_(HITP)_2_ as a dopant-free hole transport layer achieve a champion power conversion efficiency of 10.3%. This work broadens the application of metal–organic frameworks in the field of perovskite solar cells.

**Supplementary Information:**

The online version contains supplementary material available at 10.1186/s11671-021-03643-7.

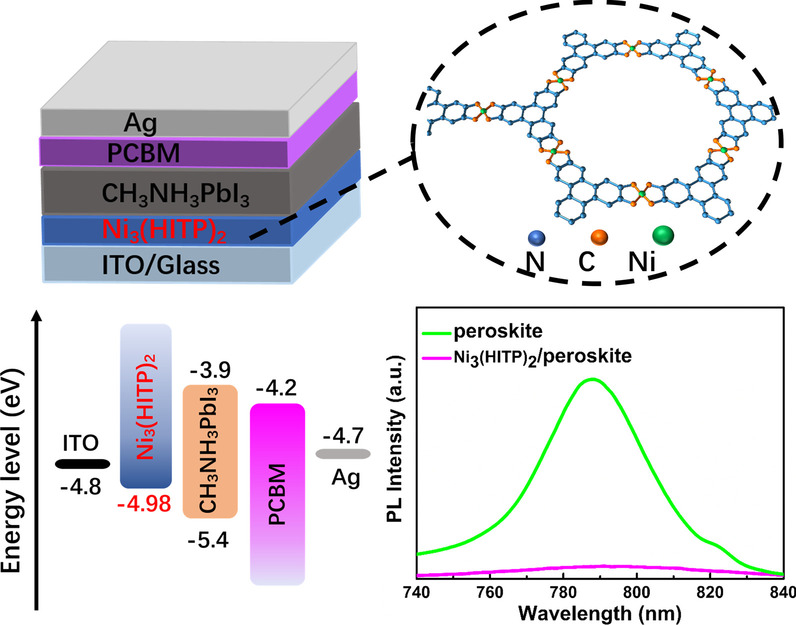

## Introduction

Organic–inorganic hybrid perovskite solar cells (PSCs) are drawing more and more attention due to its rapid upgrade of device efficiency [1–9]. So far, the highest certified power conversion efficiency (PCE) of the PSCs has reached up to 25.5% [10], approaching that of monocrystalline silicon-based solar cells. PSCs are thin-film devices, and the perovskite light active layer is sandwiched between anode and cathode. To improve the PCE and stability, the suitable hole transport layers (HTLs) are inserted between perovskite layers and anodes [11–15]. Usually, HTLs have been proven to be an important part of PSCs to reduce carrier recombination and collect holes effectively, thereby increasing open-circuit voltage and fill factor [16]. Ideal HTLs should incorporate the following desirable characteristics: (i) high carrier mobility to facilitate effective transportation holes. (ii) high stability to prolong device life. (iii) low-temperature solution process for deposition of the film.

The HTLs are divided into organic and inorganic materials. The organic HTLs have high-quality film and adjustable bandgap [17, 18]. The representative organic materials used in perovskite solar cells are poly(3,4-ethylenedioxythiophene):poly(styrene sulfonate) [19], poly[bis(4-phenyl)(2,4,6-trimethylphenyl)amine] [20], 2,2′,7,7′-tetrakis(*N*,*N*′-di-*p*-methoxyphenylamine)-9,9′-spirobiflurorene (Spiro-OMeTAD) [21] and poly(3-hexylthiophene) [22–25]. However, the hole mobility of the most organic HTLs is within 10^−2^–10^−6^ cm^2^·V^−1^·S^−1^ [26]. It limits the ability to transport holes from active layer to electrode and further restricts device efficiency. Doping is a useful method to enhance carriers mobility of organic semiconductors. For example, the hole mobility improves enormously after 4-*tert*-butyl-pyridine and bis (trifluoromethane) sulfonimide lithium salt are added into Spiro-OMeTAD. However, it also brings the problem of device instability due to the hygroscopicity of the additives, and so on [27, 28].

Traditional inorganic materials such as V_2_O_5_, Cu_2_O, MoO_3_, CuSCN, NiO_x_, and their derivatives have been widely studied due to the advantages of excellent long-term stability and high intrinsic hole mobility [29–34]. Nevertheless, most of these materials are prepared through high annealing temperature, O_2_ plasma, too time-consuming, or limited solubility. These drawbacks hinder their further development in large-scale applications and flexible devices. Therefore, it is necessary to find new HTLs with high mobility, low-temperature process, and high stability.

Metal–organic frameworks (MOFs) possess properties of high degree flexibility, including adjustable electrical [35], optical [35], and mechanical properties [36, 37]. It has attracted much attention in the fields of electronic devices [38, 39], such as memristors, field-effect transistors, supercapacitors [40], and various sensor architectures [41–43]. In recent years, MOFs have been applied in PSCs due to the properties of regular micro-pore structures and low-temperature process [44–48]. Vinogradov et al. first reported the TiO_2_-MOF-based solar cells with an efficiency of 6.4% [49]. Utilizing the typical micro-pore structure of MOFs, Ho et al. introduced MOF-525 (Zr_6_O_4_(OH)_4_(TCPP-H_2_)_3_) as the regular scaffold into perovskite film to mediate the arrangement of perovskite crystallites. Finally, they improved the morphology and crystallinity of the perovskite thin film [50]. Wei and coworkers used zeolitic imidazolate framework-8 as an interface layer to increase the crystallinity and grain size of perovskite film [51]. Fan et al. doped [In_2_(phen)_3_Cl_6_]·CH_3_CN·2H_2_O into HTLs to enhance light absorption and reduce the pinholes of the film [52]. These works improved PSCs performance effectively by regulating the morphology and crystallinity of perovskite film via adding MOFs. However, to our knowledge, MOFs as dopant-free HTLs in PSCs have not been reported. In recent years, the emergence of electrically conductive MOFs provides new opportunities for their integration as electroactive components in electronic devices [53].

Herein, we firstly attempt Ni_3_(2,3,6,7,10,11-hexaiminotriphenylene)_2_ (Ni_3_(HITP)_2_) as dopant-free HTLs in PSCs to extract holes effectively for PSCs. The Ni_3_(HITP)_2_ is a p-type semiconductor material with a high hole mobility of 48.6 cm^2^·V^−1^·s^−1^ [54], and the Ni_3_(HITP)_2_ film can be synthesized in a low-temperature process. The thickness controllable floating film of Ni_3_(HITP)_2_ at the gas–liquid interface is transferred onto indium tin oxide (ITO)-coated glass substrate. The film possesses low surface roughness, which provides prerequisites for subsequent deposition of high-quality perovskite films. Steady-state photoluminescence (PL) spectrum shows Ni_3_(HITP)_2_ film can transport holes effectively from perovskite layer to anode. As a result, the inverted planar PSCs based on Ni_3_(HITP)_2_ film achieve the champion PCE of 10.3%.

## Results and Discussion

**Fig. 1:**
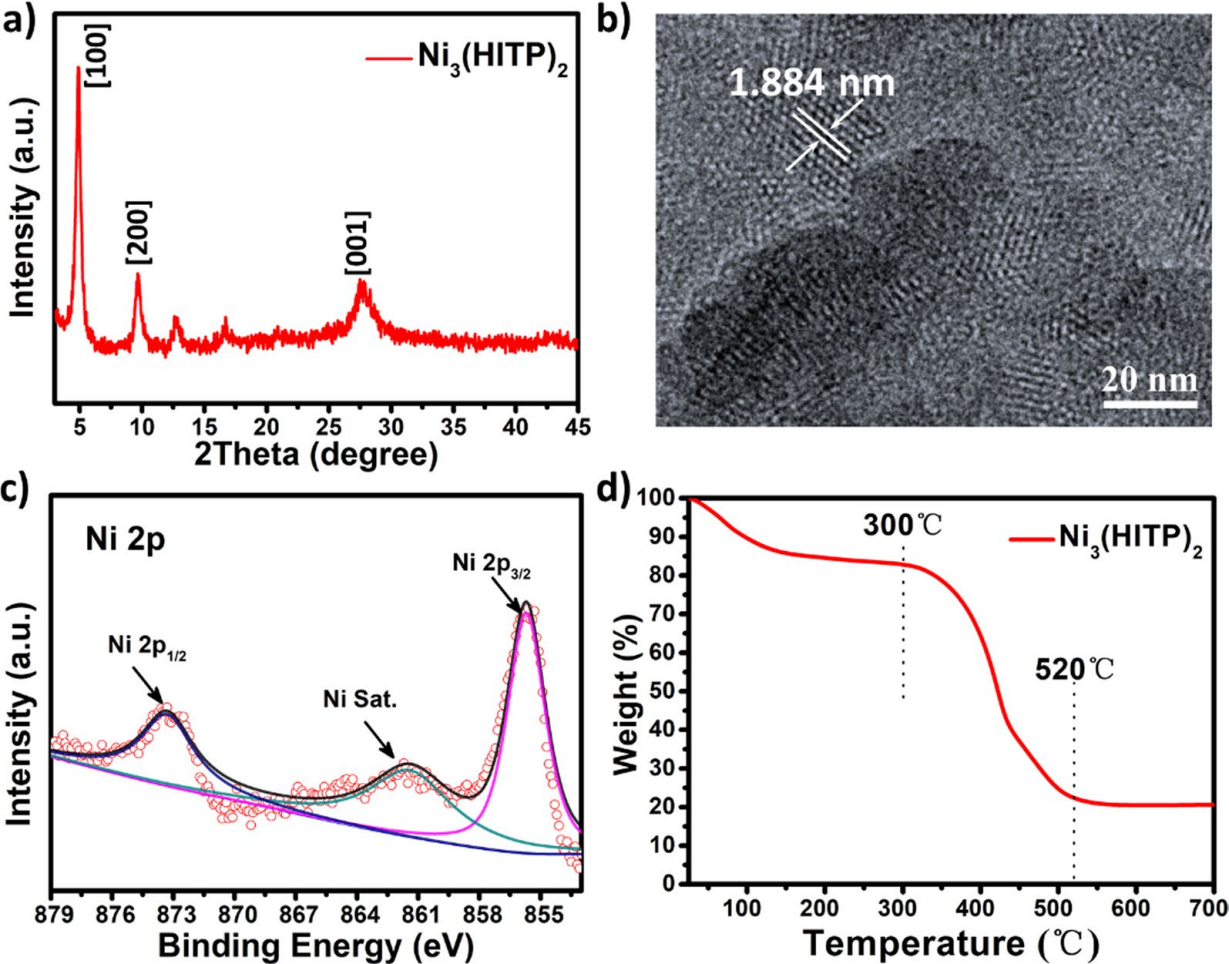
Characterization of the Ni_3_(HITP)_2_ film. **a** XRD pattern, **b** TEM micrograph; **c** XPS spectra of Ni 2p, and **d** thermogravimetric curve of the Ni_3_(HITP)_2_

The Ni_3_(HITP)_2_ film is transferred by the following method, and experimental details are provided in the experimental section and Additional file 1: Figure S1. After the reaction mixture is heated to 65 °C, a bluish film spontaneously spreads out and forms at the liquid–air interface because of the hydrophobic of the Ni_3_(HITP)_2_ film. Then, the ITO-coated glass substrate is placed at the air–liquid interface along the edge of the beaker in an inclined posture under the film. The side of the Ni_3_(HITP)_2_ film contact with water directly adsorbs on the ITO-coated glass substrate by homeopathically and slowly lifting. Finally, a complete Ni_3_(HITP)_2_ film is obtained. Figure 1a shows X-ray diffraction (XRD) characterization. The peaks of the XRD spectrum are located at 4.7°, 9.5°, 12.6°, 16.5°, and 27.3°. The peaks of 4.7°, 9.5°, 12.6°, and 16.5° correspond to the (100) reflections, and 27.3° originates from (001) reflection. The result is consistent with the structure of Ni_3_(HITP)_2_ reported in the previous literature [54]. The transmission electron microscope (TEM) result in Fig. 1b displays that the film is highly oriented and uniform without curling on the edges of these nanosheets. The Ni_3_(HITP)_2_ has a fringe spacing of 1.884 nm, corresponding to the (100) plane [55]. The energy-dispersive spectroscopy mapping images (Additional file 1: Figure S2) reveal the uniform element distribution of Ni, C, and N throughout the whole Ni_3_(HITP)_2_ film. X-ray photoelectron spectroscopy (XPS) (Additional file 1: Figure S3) is further carried out to identify the formation of Ni_3_(HITP)_2_ film. As shown in Fig. 1c, there are no other impurities such as NiO (853.8 eV) and Ni(OH)_2_ (855.2 eV) in the membrane, according to the previous reports [21]. The peaks of Ni 2p are located at 873.4 eV (2p_1/2_) and 855.5 eV (2p_3/2_) from Ni_3_(HITP)_2_ [54]. In addition, the thermogravimetric analysis is conducted to investigate the stability of the Ni_3_(HITP)_2_ film. As can be seen from Fig. 1d, water in the film is lost at the initial stage. As the temperature increases, Ni_3_(HITP)_2_ decomposes between 300 ℃ and 520 ℃. The high thermal stability provides wider application compared with organic materials.

**Fig. 2:**
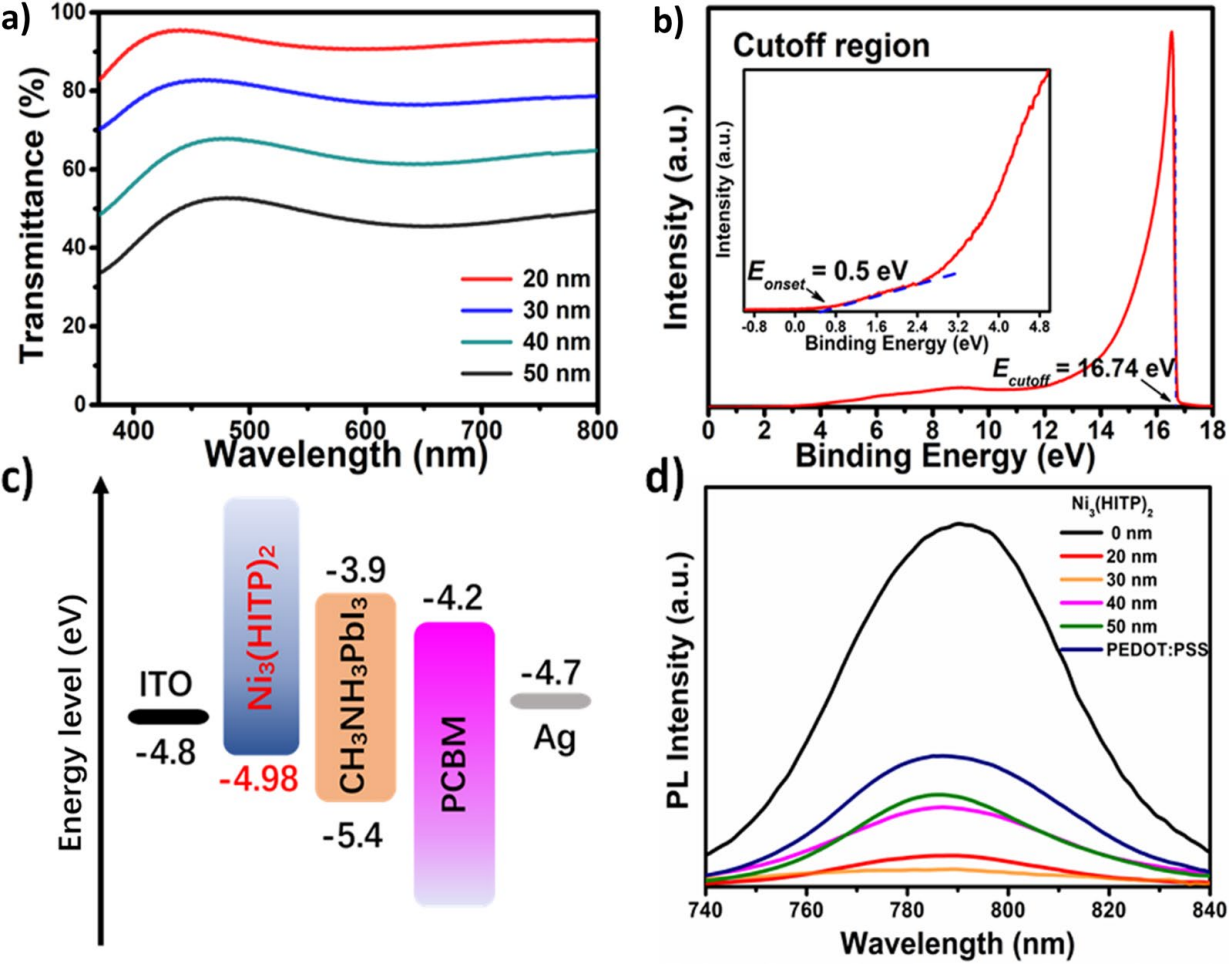
Characterization of optical and electrical properties of Ni_3_(HITP)_2_ film. **a** Optical transmission spectra of different thickness of Ni_3_(HITP)_2_ films; **b** UPS spectra of Ni_3_(HITP)_2_ film; **c** Energy level alignment diagram of PSCs and **d** The steady-state PL spectra of perovskite films on different thickness Ni_3_(HITP)_2_ films

By controlling the reaction time, we obtained the different thickness films (Additional file 1: Figure S4). The Ni_3_(HITP)_2_ film gradually changes from light blue to bluish-black or even black as the film thickness increases (Additional file 1: Figure S5). Figure 2a shows the optical transmittance of Ni_3_(HITP)_2_ films with different thicknesses. The transmittance of these films decreases with increasing film thickness. The films with thickness of 20 and 30 nm maintain over 75% transmittance. The hole mobility of the Ni_3_(HITP)_2_ films reaches up to 48.6 cm^2^·V^−1^·s^−1^, and it is higher than that of most hole transport materials and even some inorganic materials. The high carrier mobility is conducive to hole transportation in photo-electronic devices [56]. The electronic properties of the Ni_3_(HITP)_2_ film are further conducted by ultraviolet photoemission spectroscopy (UPS) (Fig. 2b). The Femi level (*E*_f_) of 4.48 eV and valence band maximum (*V*_B_) of 4.98 eV are obtained from the secondary electron cutoff and the onset of the UPS spectra according to the following equations: *E*_f_ = *hv* − *E*_cutoff_ and *V*_B_ = *hv *− (*E*_cutoff_ − *E*_onset_), where *hv* is the incident photon energy of the He (I) source (21.22 eV). Figure 2c shows the energy level alignment diagram of the inverted PSCs. The result demonstrates that the *V*_B_ of Ni_3_(HITP)_2_ and perovskite (~ 5.4 eV) are matched well. It indicates the Ni_3_(HITP)_2_ film is desirable to act as HTL for PSCs. Steady-state PL spectra is performed to ascertain the hole transfer ability from perovskite film to Ni_3_(HITP)_2_ layer (Fig. 2d). After introducing PEDOT/PSS, the perovskite films show strong PL quenching, indicating the holes are transferred from perovskite to HTL of PEDOT/PSS. The PL of perovskite film is further quenched, when Ni_3_(HITP)_2_ replaces PEDOT/PSS. Especially, 30 nm Ni_3_(HITP)_2_ film leads to the lowest PL intensity, suggesting more carriers are transferred effectively from perovskite to HTL. In addition, we have repeated the PL of 20 nm and 30 nm Ni_3_(HITP)_2_ for three times to compare with other thicknesses of Ni_3_(HITP)_2_ (Additional file 1: Figure S6). The graphs marked with the red box are the PL of perovskite with 20 nm and 30 nm Ni_3_(HITP)_2_ and are further amplified in the inset of Additional file 1: Figure S6. The time-resolved PL measurement is carried out to analyze the hole extraction capability of Ni_3_(HITP)_2_ film (Additional file 1: Figure S7). The average decay lifetimes of the perovskite deposited on ITO substrate with different hole transport layers are listed in Additional file 1: Table S1. Compared with PEDOT/PSS, the average carriers lifetime of perovskite drops greatly upon introducing Ni_3_(HITP)_2_ film, indicating that the holes can efficiently be extracted at the interfaces of perovskite and Ni_3_(HITP)_2_. It is worthy of noting that the shorter decay lifetimes perovskite based on the 30 nm Ni_3_(HITP)_2_ film declines to 1.18 ns, revealing its high carrier extraction capability.

**Fig. 3:**
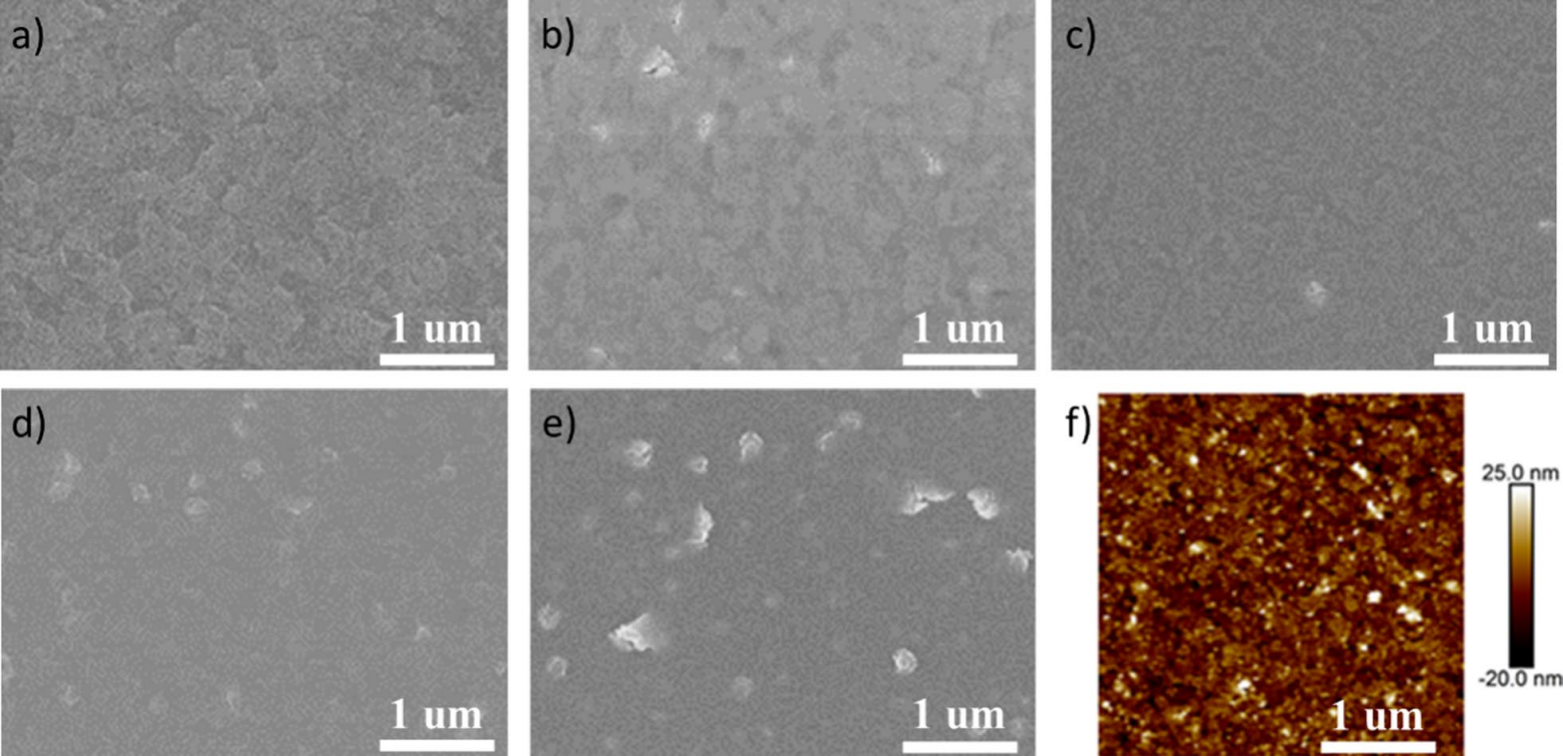
Morphology characterization of Ni_3_(HITP)_2_ films with different thicknesses. SEM images of **a** Bare ITO-coated glass and **b**–**e** Ni_3_(HITP)_2_ films with 20 nm, 30 nm, 40 nm, 50 nm, respectively; **f** AFM image of Ni_3_(HITP)_2_ film of 30 nm thickness

To investigate the surface quality of the Ni_3_(HITP)_2_ films with different thicknesses, scanning electron microscopy (SEM) images are exhibited in Fig. 3. Compared with the ITO-coated glass substrate, both 20 nm and 30 nm thickness of the Ni_3_(HITP)_2_ films remain the similar state of ITO, manifesting highly transparent property. Further increasing the thickness of films, the surface morphology character of the ITO disappears. Meanwhile, the film becomes non-uniform and has some white spots. Figure 3f and Additional file 1: Figure S8 show the surface morphology of Ni_3_(HITP)_2_ films by atomic force microscopy (AFM). The root-mean-square (RMS) roughness is 9.74 nm for the Ni_3_(HITP)_2_ films with a thickness of 20 nm. When increasing to 30 nm thickness, RMS roughness increases to 5.5 nm. Nevertheless, further increasing the thickness of Ni_3_(HITP)_2_ films, the film surface becomes rougher with RMS roughness of 14.2 nm and 16.3 nm for 40 nm and 50 nm thickness of Ni_3_(HITP)_2_ films, respectively. The results of AFM and SEM show that the Ni_3_(HITP)_2_ film with a thickness of 30 nm has a smooth and compact surface. It is the guarantee for subsequent deposition of high-quality perovskite film for solar cells.

**Fig. 4:**
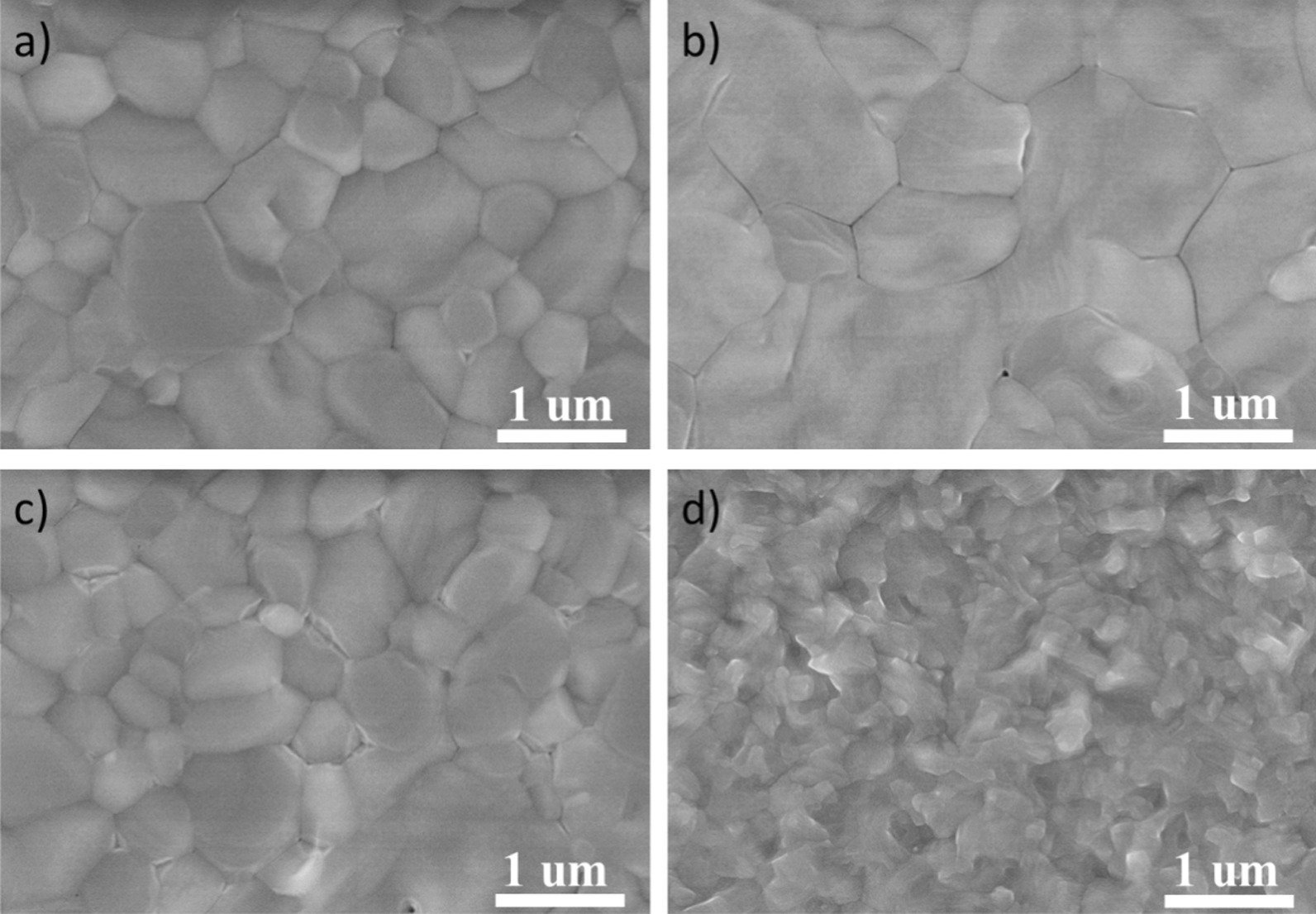
Morphology characterization of perovskite films deposition on different thicknesses of Ni_3_(HITP)_2_ films. **a**–**d** 20 nm, 30 nm, 40 nm, and 50 nm, respectively

To investigate the morphology and crystallinity of the perovskite films on Ni_3_(HITP)_2_ films, Fig. 4 and Additional file 1: Figure S9 show the surface SEM images and XRD pattern of perovskite film. The perovskite layer is prepared by a two-step method, which avoids Ni_3_(HITP)_2_ film being corroded by the solvent N, N-dimethyl formamide and dimethyl sulfoxide. As can be seen from Fig. 4, all the perovskite films based on different thicknesses of Ni_3_(HITP)_2_ films have compact surfaces, but they still have obvious differences. The grain boundary of perovskite film deposited on the 20 nm Ni_3_(HITP)_2_ film is clearly visible. When increasing the thickness of the Ni_3_(HITP)_2_ film to 30 nm, the perovskite grain boundaries gradually become blurred. It indicates the perovskite grains are closely packed together. Meanwhile, the perovskite grain size is increased to 2 um, contributing to the smoother film surface of Ni_3_(HITP)_2_ film. When further increasing the thickness of Ni_3_(HITP)_2_ film, the perovskite grains become smaller, and the perovskite film surface gets uneven. Furthermore, Additional file 1: Figure S9 further shows the quality of perovskite films deposition on different thicknesses of Ni_3_(HITP)_2_ membranes. It can be seen that the XRD peak intensity of the perovskite deposited on the surface of the 30 nm-thick Ni_3_(HITP)_2_ film is higher than that of other thicknesses of perovskite films. The results demonstrate the perovskite film with 30 nm Ni_3_(HITP)_2_ has the highest crystallinity.

**Fig. 5:**
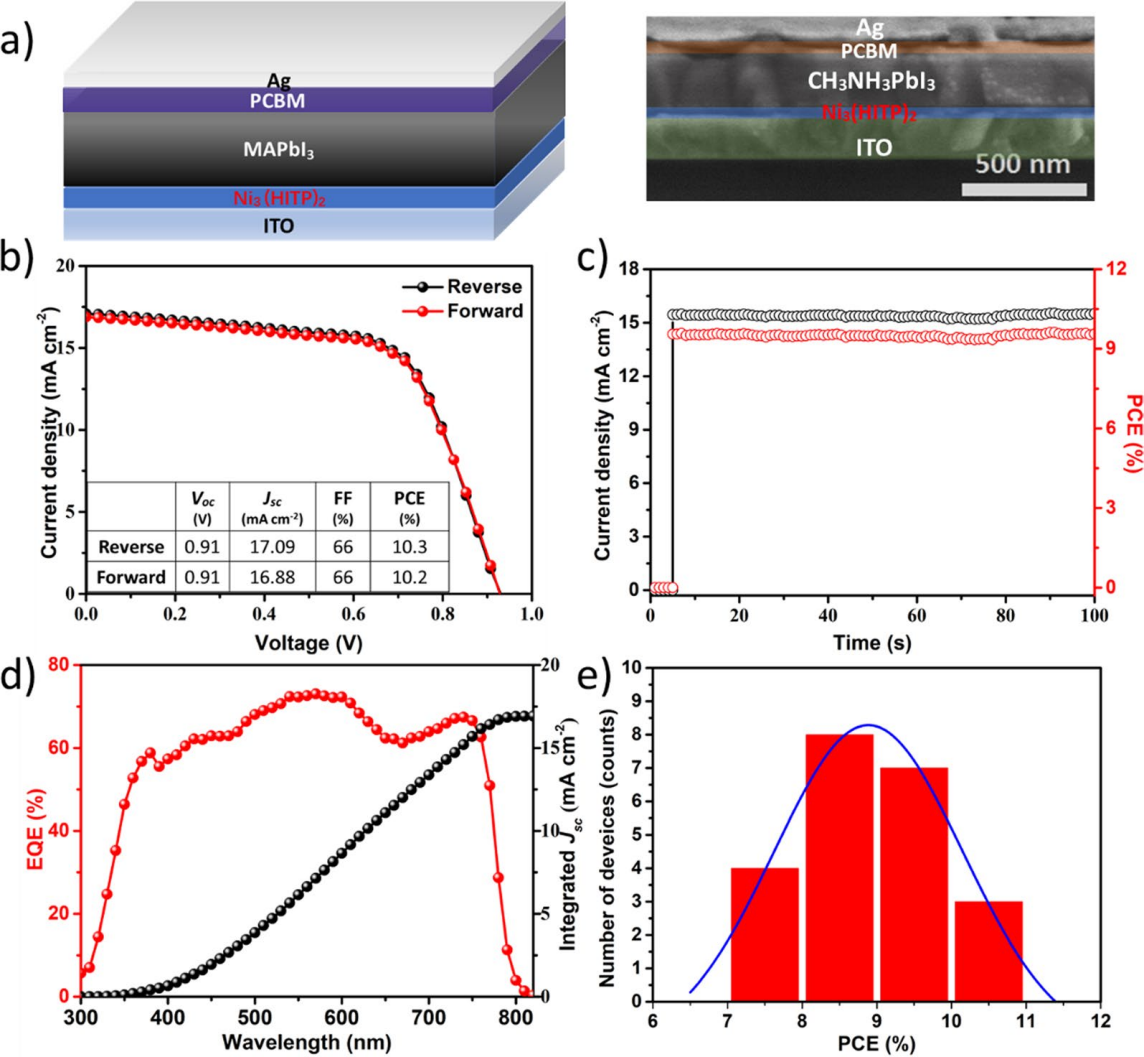
The characterization of the PSCs. **a** The device structure and SEM image of cross section; **b** the *J*–*V* curves of the device under forward scan and reverse scan; **c** steady-state photocurrent (black curve) and output efficiency (red curve) of the device; **d** EQE and corresponding integrated *J*_sc_; **e** histogram of PCEs measured from 22 PSCs

To build up a good performance solar cell, the Ni_3_(HITP)_2_ films with a thickness of 30 nm are used to fabricate the p-i-n type inverted PSCs due to its high transmittance, good flatness, high hole mobility, and appropriate energy level. Figure 5a shows the device has clear layers from the cross-sectional view and the thickness of perovskite is about 300 nm. The *J*–*V* measurement of PSCs is conducted under the standard AM 1.5G illumination. As shown in Fig. 5b, the device exhibits a negligible photocurrent hysteresis under different scanning directions [57, 58]. The champion device has a PCE of 10.3%, *V*_oc_ of 0.91 V, *J*_sc_ of 17.09 mA·cm^−2^, and FF of 66%. Figure 5c shows the steady-state photocurrent density and efficiency evolved with time at the maximum power output point (0.75 V). A reliable output efficiency of 9.61% and photocurrent density of 15.45 mA·cm^−2^ are obtained. Figure 5d shows the external quantum efficiency (EQE) of the device. The integrated *J*_sc_ from the EQE spectrum is 16.94 mA·cm^−2^, which is consistent with the value from the *J*–*V* curve. The EQE of the device is no more than 73% at the wavelength between 400—800 nm. To overcome the problem, the Ni_3_(HITP)_2_ film is inserted between perovskite film and top electrode will be a good strategy. This kind of work is underway. In addition, Fig. 5e illustrates the good reproducibility of the devices with the PCE histogram collected from 22 independent devices.

## Conclusions

In summary, a dopant-free Ni_3_(HITP)_2_ endows the suitable valence band edge and high hole mobility as HTLs for PSCs. The steady-state and time-resolved PL spectrum exhibit high hole extraction capability of Ni_3_(HITP)_2_. Inverted PSCs based on Ni_3_(HITP)_2_ films have a champion PCE of 10.3%. This work fills the gap in the application of MOFs as dopant-free hole transport layers in PSCs and expands the application field of MOFs.

## Materials and Methods

### Materials

All the chemicals were bought from commercial resources without additional purification. Water was purified with the Milli-Q purification system. Nickel chloride hexahydrate and ammonium hydroxide were bought from Sinopharm Chemical Reagent Co. 2,3,6,7,10,11-hexaaminotriphenylene hexahydrochloride was bought from WuXi AppTec. Lead (II) iodide was obtained from Sigma-Aldrich.

### *Synthesis of the Ni*_*3*_*(HITP)*_*2*_* Film*

20 mg of 2,3,6,7,10,11-hexaaminotriphenylene hexahydrochloride, 13.2 mg of nickel chloride hexahydrate, and 40.0 mL H_2_O were added into a 50-mL beaker. The reaction mixture was then sonicated until the solids were completely dissolved. After that, 0.6 mL ammonium hydroxide was dropped into the beaker when the reaction mixture was heated to 65 °C. The Ni_3_(HITP)_2_ film was formed at the air–liquid interface after 1 min. Different thickness of the films was controlled by the reaction time.

### Device Fabrication

ITO-coated glass substrates were sequentially cleaned by sonication with acetone, deionized water, and ethyl alcohol and then were treated using UV-ozone. The processed ITO glass was extended to the bottom of the film along the edge of the beaker in an inclined posture. The complete Ni_3_(HITP)_2_ film was obtained by homeopathically and slowly lifted onto ITO-coated glass substrate. Then, after further cleaning and drying the Ni_3_(HITP)_2_ film adsorbed on ITO-coated glass substrate, the perovskite layer was fabricated as described in our previous report [57]. The electron-transporting layer, PC_61_BM (methyl [6, 6]-phenyl-C_61_-butyrate) (20 mg/mL in chlorobenzene) was deposited by spin-coating. Finally, the Ag electrode was thermally evaporated in a high vacuum chamber through a metal mask. The device’s effective area was 0.0725 cm^2^.

### Characterization

The morphologies images were obtained using AFM (Bruker) and SEM (Hitachi SU8010). The TEM images were obtained by using FEI Tecnai F-20 microscope equipped with a field-emission gun (operating at 200 kV). The transmission spectra of the films were recorded by the Shimadzu spectrophotometer (mode UV2450) for PL measurements were performed by a Horiba spectrofluorometer (Fluoromax-4). The wavelength of the excitation light source is 525 nm. XRD and TGA measurements were carried out on the D8 Advance (Bruker) and TG/DTG7300 (SII NanoTechnology), respectively. XPS and UPS were performed by the Escalab 250Xi (Thermo Fisher). The *J*–*V* characteristics of devices were recorded from a programmable Keithley 2400 source meter under simulated AM 1.5G solar irradiation at 100 mW·cm^−2^ in air condition.

## Supplementary Information

**Additional file 1: Figure S1**. Illustration of the assembly process for the Ni_3_(HITP)_2_ film. **Figure S2**. EDX mapping of the Ni_3_(HITP)_2_ membranes. **Figure S3**. XPS spectra of the Ni_3_(HITP)_2_ membranes. **Figure S4**. AFM images of different thicknesses of Ni_3_(HITP)_2_ membranes. **Figure S5**. Photographs of the bare ITO glass and with different thicknesses of Ni_3_(HITP)_2_ membranes. **Figure S6**. Steady-state PL spectra of perovskite/ITO based on 20 nm, 30 nm, 40 nm, and 50 nm Ni_3_(HITP)_2_ film and PEDOT/PSS; Inset: the enlarged steady-state PL spectra of perovskite on the 20 nm and 30 nm Ni_3_(HITP)_2_ film repeated by three times, respectively. **Figure S7**. Time-resolved PL spectra of perovskite on the ITO substrate with different thicknesses of Ni_3_(HITP)_2_ film and PEDOT/PSS. **Figure S8**. AFM images of different thicknesses of Ni_3_(HITP)_2_ membranes. **Figure S9**. XRD pattern of perovskite films deposition on different thicknesses of Ni_3_(HITP)_2_ membranes. **Table S1**. The average decay lifetimes of the perovskite/ITO with different hole transport layers.

## Data Availability

All data supporting the conclusions of this article are included within the article and supplementary document.

## Abbreviations

PSCs: Perovskite solar cells
PCE: Power conversion efficiency
ITO: Indium tin oxide
HTLs: Hole transport layers
Spiro-OMeTAD: 2,2′,7,7′-Tetrakis(*N*,*N*′-di-*p*-methoxyphenylamine)-9,9′-spirobiflurorene
MOFs: Metal–organic frameworks
Ni_3_(HITP)_2_: Ni_3_(2,3,6,7,10,11-hexaiminotriphenylene)_2_
PL: Steady-state photoluminescence
EQE: External quantum efficiency
XRD: X-ray diffraction
TEM: Transmission electron microscope
XPS: X-ray photoelectron spectroscopy
UPS: Ultraviolet photoemission spectroscopy
*E*_f_: Femi level
*V*_B_: Valence band maximum
SEM: Scanning electron microscopy
AFM: Atomic force microscopy
RMS: Root-mean-square
