# Supplementary material for: High-Hole-Mobility Metal–Organic Framework as Dopant-Free Hole Transport Layer for Perovskite Solar Cells
Source: Nanoscale Res Lett. 2022 Jan 4;17:6. doi: 10.1186/s11671-021-03643-7 (PMC8738790; doi:10.1186/s11671-021-03643-7)
Supplement: Supplementary file 1 — Additional file 1: Figure S1. Illustration of the assembly process for the Ni3(HITP)2 film. Figure S2. EDX mapping of the Ni3(HITP)2 membranes. Figure S3. XPS spectra of the Ni3(HITP)2 membranes. Figure S4. AFM images of different thicknesses of Ni3(HITP)2 membranes. Figure S5. Photographs of the bare ITO glass and with different thicknesses of Ni3(HITP)2 membranes. Figure S6. Steady-state PL spectra of perovskite/ITO based on 20 nm, 30 nm, 40 nm, and 50 nm Ni3(HITP)2 film and PEDOT/PSS; Inset: the enlarged steady-state PL spectra of perovskite on the 20 nm and 30 nm Ni3(HITP)2 film repeated by three times, respectively. Figure S7. Time-resolved PL spectra of perovskite on the ITO substrate with different thicknesses of Ni3(HITP)2 film and PEDOT/PSS. Figure S8. AFM images of different thicknesses of Ni3(HITP)2 membranes. Figure S9. XRD pattern of perovskite films deposition on different thicknesses of Ni3(HITP)2 membranes. Table S1. The average decay lifetimes of the perovskite/ITO with different hole transport layers. [file 11671_2021_3643_MOESM1_ESM.docx]

**High-Hole-Mobility Metal-Organic Framework as Dopant-Free Hole Transport Layer for****Perovskite Solar Cells**

**Ruonan Wang,^a‡^ Weikang Yu,^a,b‡^ Cheng Sun,^a‡^ Kashi Chiranjeevulu,^c^ Shuguang Deng,^d^ Jiang Wu,^e^ Feng Yan,^f^ Changsi Peng,^g^ Yanhui Lou,*^,a^ Gang Xu,*^,c^ Guifu Zou*^,a^**

*^a^ College of Energy, Soochow Institute for Energy and Materials Innovations, and Key Laboratory of Advanced Carbon Materials and Wearable Energy Technologies of Jiangsu Province, Soochow University, Suzhou 215123, China.*

*^b^ School of Resources Environmental & Chemical Engineering, Nanchang University, 999 Xuefu Avenue, Nanchang 330031, China.*

*^c^ State Key Laboratory of Structural Chemistry, Fujian Institute of Research on the Structure of Matter, Chinese Academy of Sciences, Fuzhou, Fujian 350002, China.*

*^d^ School for Engineering of Matter, Transport and Energy, Arizona State University, 551 E. Tyler Mall, Tempe, AZ 85287, USA.*

*^e^ Institute of Fundamental and Frontier Sciences, University of Electronic Science and Technology of China, Chengdu 610054, P. R. China.*

*^f^ College of Chemistry, Chemical Engineering and Materials Science, Soochow Universit, Suzhou 215123 (China).*

*^g^ School of Optoelectronic Science and Engineering and Collaborative Innovation Center of Suzhou Nano Science and Technology, Soochow University, Suzhou, 215006, China.*

***^‡^ These three authors contributed equally to the work.***

******* Email: [yhlou@suda.edu.cn](mailto:yhlou@suda.edu.cn); [gxu@fjirsm.ac.cn](mailto:gxu@fjirsm.ac.cn); [zouguifu@suda.edu.cn](mailto:zouguifu@suda.edu.cn)

**Content:**

**Supporting figures**

**
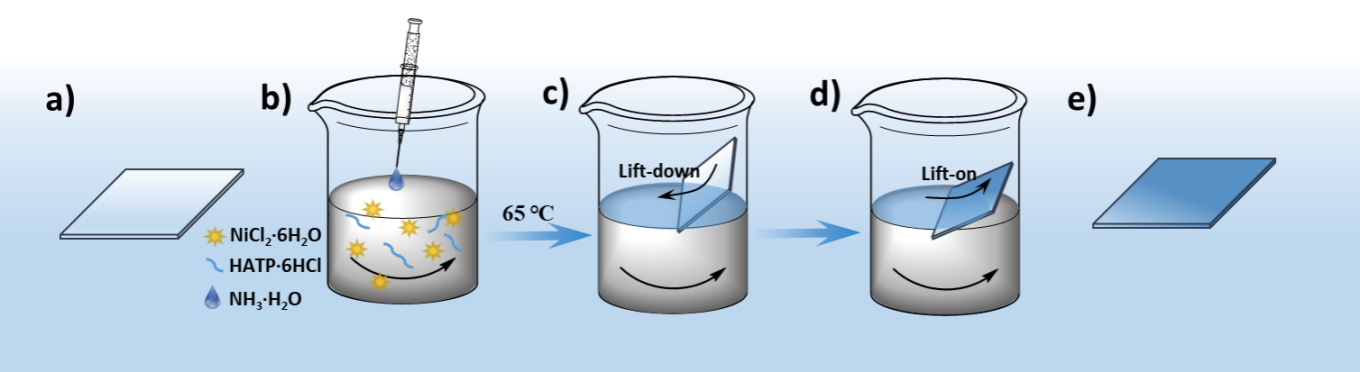
**

**Figure S1.** Illustration of the assembly process for the Ni_3_(HITP)_2_ film. a) The cleaned ITO substrate; b) The precursor solution for synthesis of Ni_3_(HITP)_2_ film; c) After the Ni_3_(HITP)_2_ film is formed, lift-down ITO along of the beaker; d) Lift-on the membrane by slowly raising the ITO and e) ITO substrate coated with complete Ni_3_(HITP)_2_ film.

**
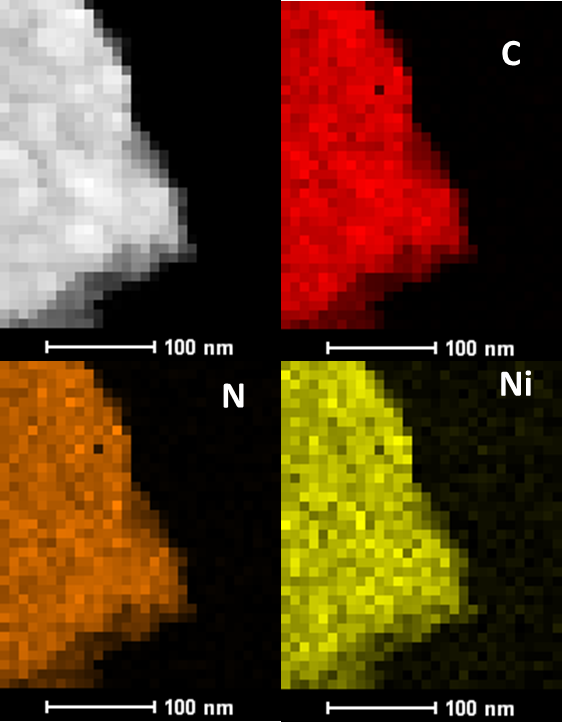
**

**Figure S2.** EDX mapping of the Ni_3_(HITP)_2_ membranes.


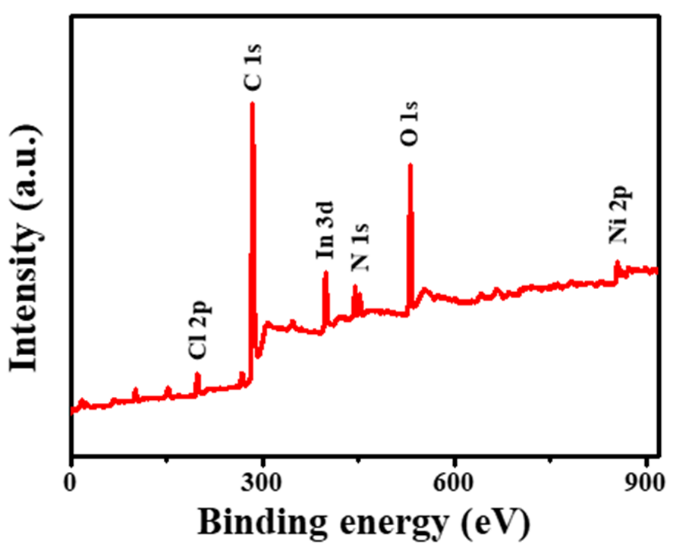
**Figure S3.** XPS spectra of the Ni_3_(HITP)_2_ membranes.


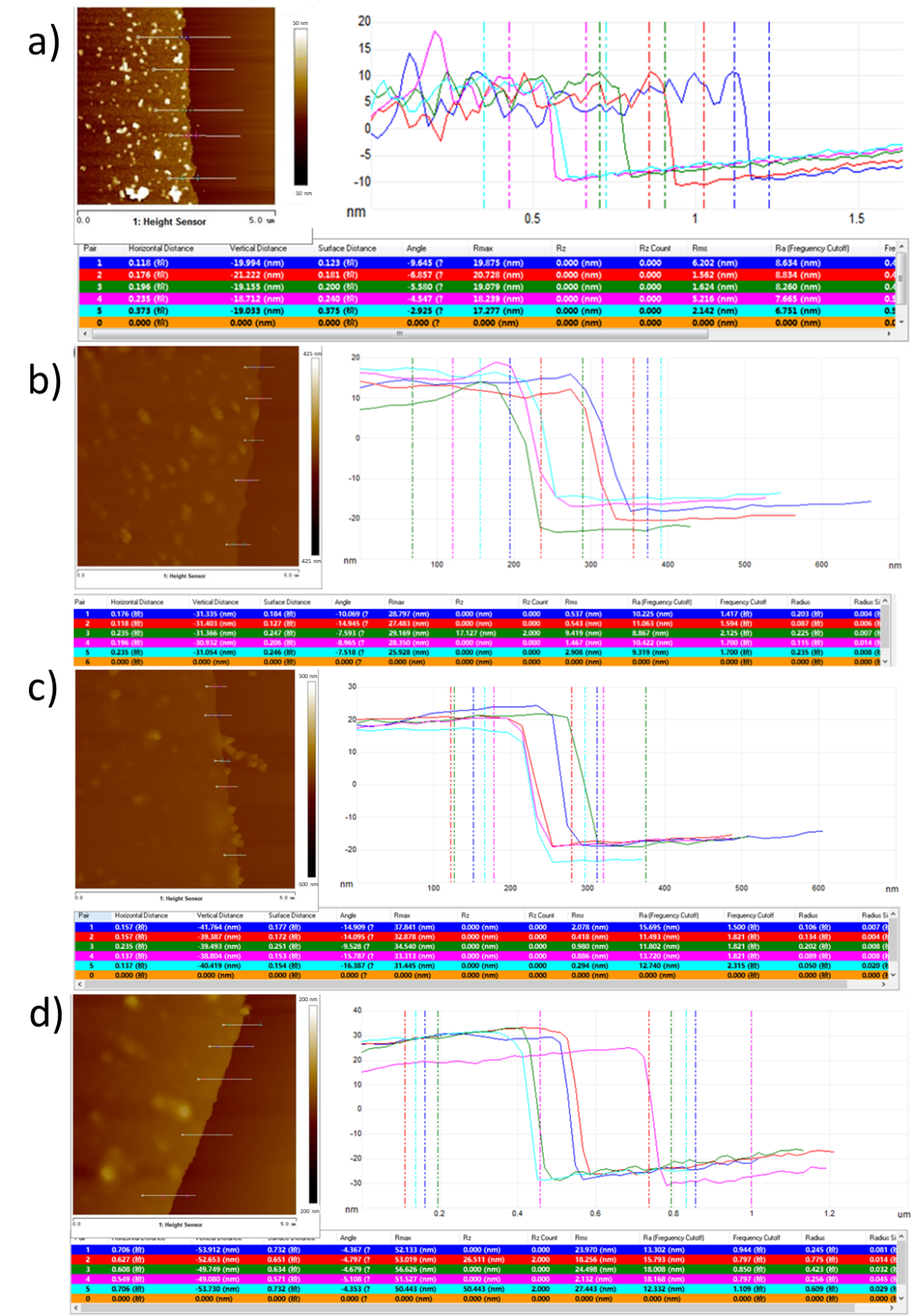


**Figure S4.** AFM images of different thickness of Ni_3_(HITP)_2_ membranes.

**
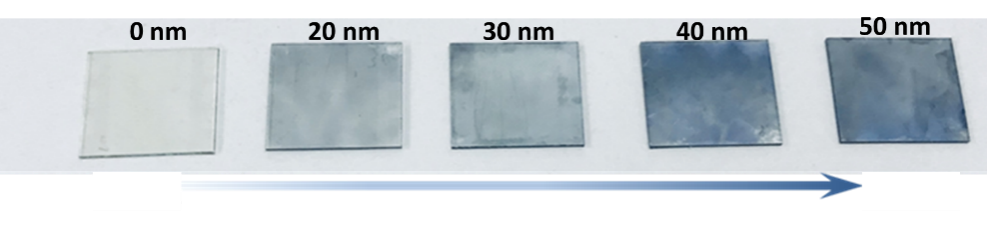
**

**Figure S5.** Photographs of the bare ITO glass and with different thickness of Ni_3_(HITP)_2_ membranes.


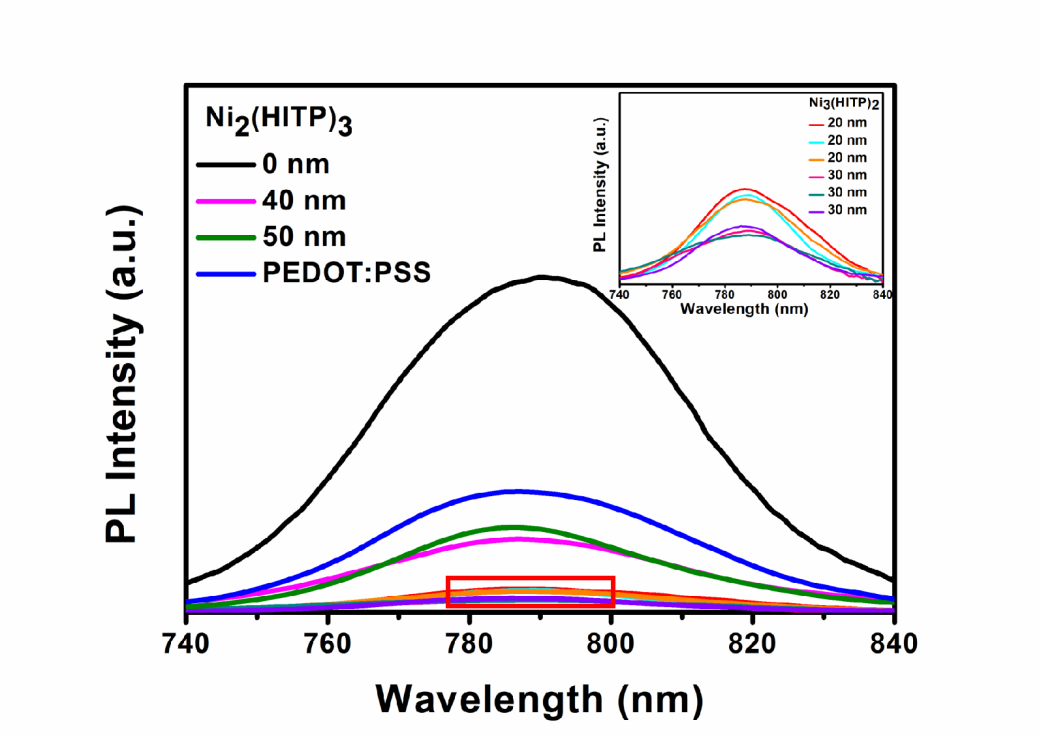


**Figure S6.** Steady-state PL spectra of perovskite/ITO based on 20nm, 30nm, 40nm, and 50nm Ni_3_(HITP)_2_ film and PEDOT/PSS; Inset: the enlarged steady-state PL spectra of perovskite on the 20nm and 30nm Ni_3_(HITP)_2_ film repeated by three times, respectively.





**Figure S7.** Time-resolved PL spectra of perovskite on the ITO substrate with different thicknesses of Ni_3_(HITP)_2_ film and PEDOT/PSS.

**
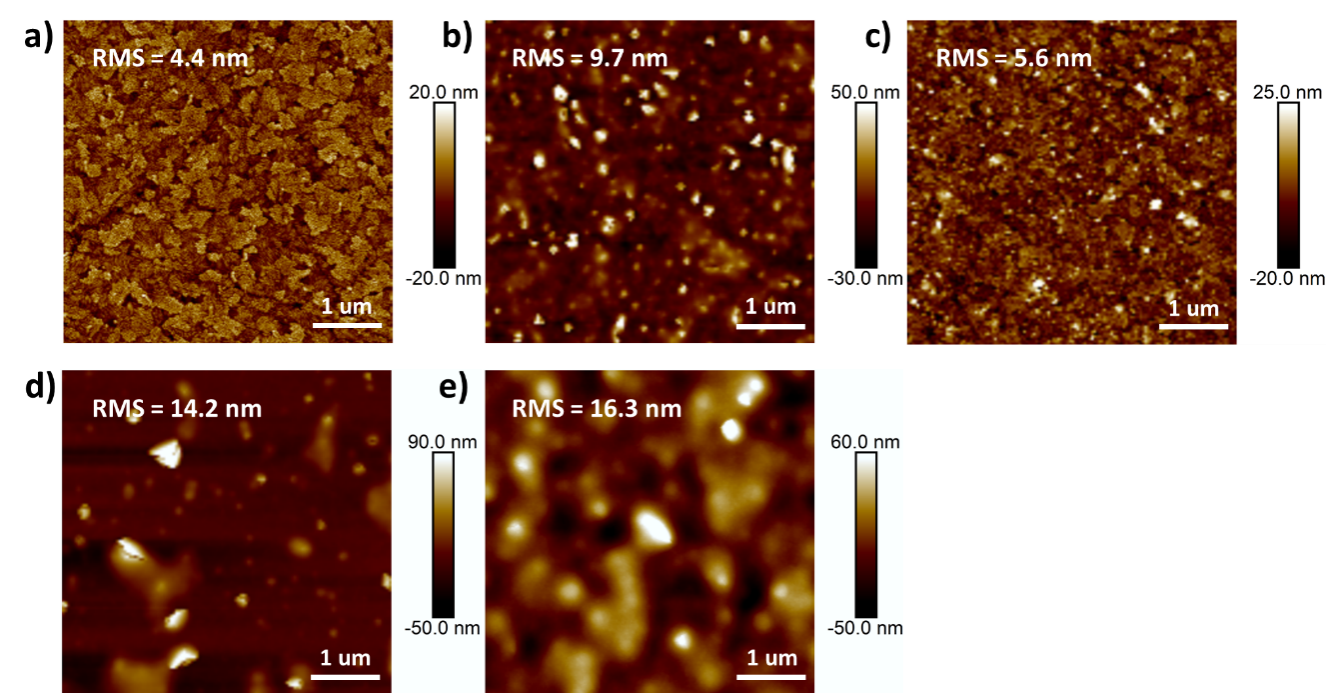
Figure S8.** AFM images of different thickness of Ni_3_(HITP)_2_ membranes. a) Bare ITO glass, b)-e) Corresponding to 20 nm, 30 nm, 40nm and 50 nm Ni_3_(HITP)_2_ membranes, respectively.


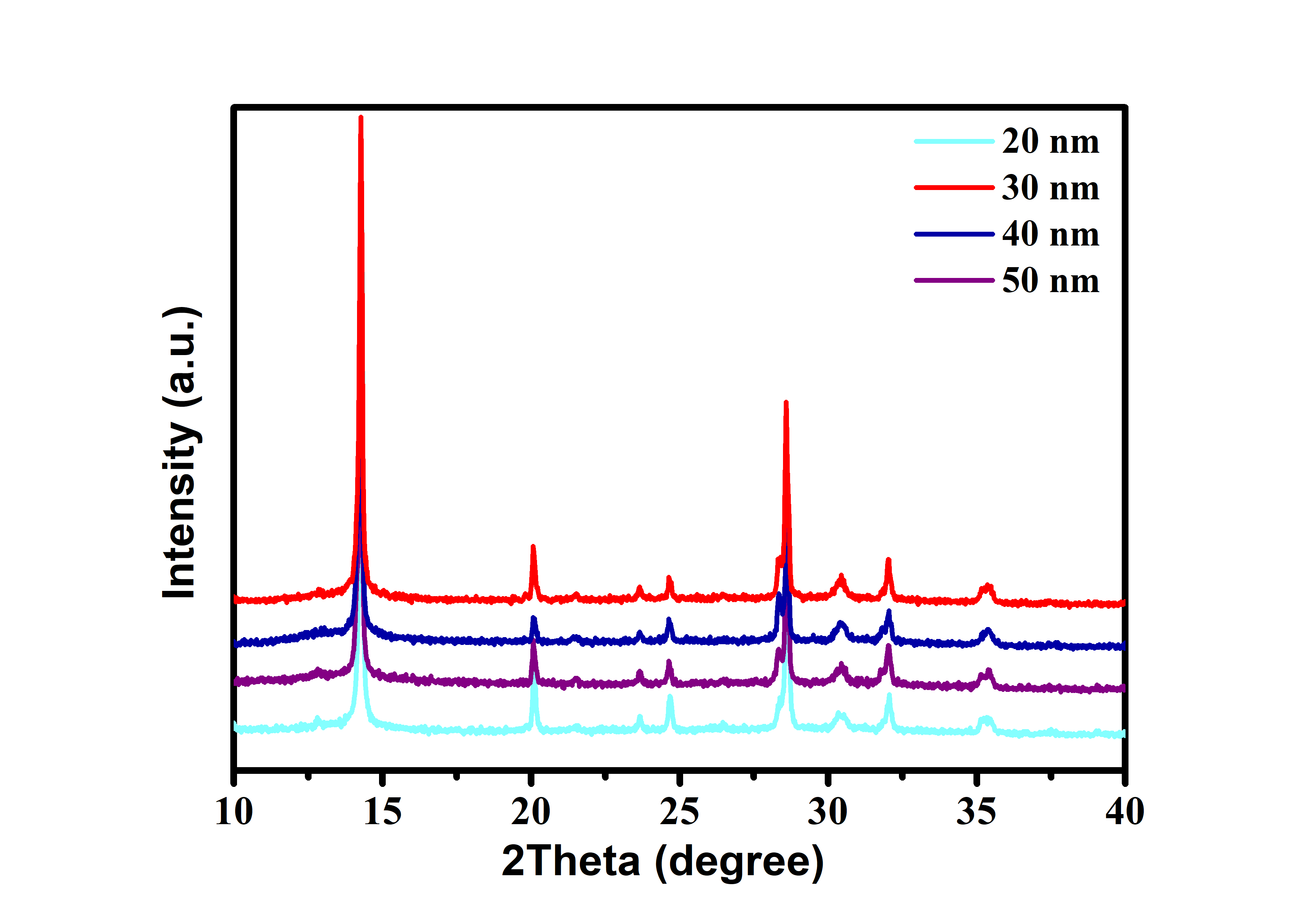


**Figure S9.** XRD pattern of perovskite films deposition on different thickness of Ni_3_(HITP)_2_ membranes.

**Table S1.** The average decay lifetimes of the perovskite/ITO with different hole transport layers.

|  | Without | PEDOT/PSS | Ni_3_(HITP)_2_ (20 nm) | Ni_3_(HITP)_2_ (30 nm) | Ni_3_(HITP)_2_ (40 nm) | Ni_3_(HITP)_2_ (50 nm) |
| --- | --- | --- | --- | --- | --- | --- |
| Average τ(ns) | 18.14 | 6.17 | 1.89 | 1.18 | 2.82 | 3.02 |
